# Characteristics and outcomes of critically ill patients with covid-19 in Sakarya, Turkey: a single centre cohort study

**DOI:** 10.3906/sag-2005-57

**Published:** 2021-04-30

**Authors:** Havva KOCAYİĞİT, Kezban ÖZMEN SÜNER, Yakup TOMAK, Gürkan DEMİR, İbrahim KOCAYİĞİT, Selçuk YAYLACI, Yusuf AYDEMİR, Mehmet KÖROĞLU, Oğuz KARABAY, Mehmet Halil ÖZTÜRK, Ali Fuat ERDEM

**Affiliations:** 1 Department of Anaesthesiology and Reanimation, Sakarya University Education and Research Hospital, Sakarya Turkey; 2 Department of Intensive Care, Sakarya University Education and Research Hospital, Sakarya Turkey; 3 Department of Anaesthesiology and Reanimation, Faculty of Medicine, Sakarya University, Sakarya Turkey; 4 Department of Cardiology, Faculty of Medicine, Sakarya University, Sakarya Turkey; 5 Department of Internal Medicine, Faculty of Medicine, Sakarya University, Sakarya Turkey; 6 Department of Pulmonary Diseases, Faculty of Medicine, Sakarya University, Sakarya Turkey; 7 Department of Medical Microbiology, Faculty of Medicine, Sakarya University, Sakarya Turkey; 8 Department of Infectious Diseases and Clinical Microbiology, Faculty of Medicine, Sakarya University, Sakarya Turkey; 9 Department of Radiology, Faculty of Medicine, Sakarya University, Sakarya Turkey

**Keywords:** Severe acute respiratory syndrome coronavirus 2 (SARS-CoV-2), coronavirus disease 2019 (Covid-19), intensive care unit

## Abstract

**Background/aim:**

Severe acute respiratory syndrome coronavirus 2 (SARS-CoV-2) was first reported in Turkey on March 10, 2020 and the number of the patients are increasing day by day. Coronavirus disease 2019 (Covid-19) has high mortality rates in intensive care units (ICUs). We aimed to describe the demographic characteristics, comorbidities, treatment protocols, and clinical outcomes among the critically ill patients admitted to the ICU of our hospital.

**Materials and methods:**

This cohort study included 103 consecutive patients who had laboratory confirmed Covid-19 and admitted to ICU of Sakarya University Training and Research Hospital between March 19 and April 13, 2020. The final date of the follow-up was April 18.

**Results:**

The mean age of the patients was 69.6 ± 14.1 years. Most of the patients had increased CRP (99%), serum ferritin (73.8%), d-dimer (82.5%), and hs-troponin levels (38.8%). 34 patients (33%) had lymphocytopenia, 24 patients (23.3%) had thrombocytopenia. 63 patients (61.2%) developed acute respiratory distress syndrome (ARDS), 31 patients (30.1%) had acute kidney injury, and 52 patients (50.5%) had multiple organ dysfunction syndrome (MODS) during follow-up. Sixty-two patients (60.2%) received mechanical ventilation. As of April 18, of the 103 patients, 52 (50.5%) had died, 30 (29.1%) had been discharged from the ICU, 21 (20.4%) were still in the ICU.

**Conclusions:**

Covid-19 has high mortality rates in ICU. Patients with elevated procalcitonin, hs-troponin, d-dimer, and CRP levels and lower platelet count at admission have higher mortality.

## 1. Introduction

Severe acute respiratory syndrome coronavirus 2 (SARS-CoV-2) was first reported in Turkey on March 10, 2020. Since the first confirmed report from Wuhan, China, more than 2,200,000 of coronavirus disease 2019 (Covid-19) cases have been confirmed worldwide [1,2]. The total number of patients with Covid-19 in Turkey is increasing day by day; as of April 18, there had been 1890 deaths and 82,329 confirmed cases. Most of the cases were reported from İstanbul, which is the largest city of Turkey [3]. Our tertiary hospital, which has 80 intensive care units (ICU) bed capacity and is the largest and the only hospital in the city of Sakarya (over 1,000,000 population) near İstanbul province, has been declared as a pandemic hospital after the confirmation of the first case in Turkey. 

Covid-19 generally has a good prognosis in many patients but older patients and those with comorbidities may have worse outcomes [4]. This condition may progress to acute respiratory distress syndrome (ARDS) and end-organ failure; thus, these patients usually need ICU follow-up. The widespread outbreak of the disease forces the ICU capacity in most countries especially in China, Italy, France, and Spain [5,6]. The accurate identification of the critically ill patients with Covid-19 is important to guide the effective use of the ICU capacity. There are conflicting results in the literature about treatment modalities and mechanical ventilator management strategies in the ICU follow-up of patients with Covid-19. In this report, we aimed to describe the demographic characteristics, comorbidities, treatment protocols, and clinical outcomes among the critically ill patients admitted to ICU of our hospital. 

## 2. Methods

### 2.1. Study population

This cohort study included 103 consecutive patients who had laboratory confirmed Covid-19 and presented to the ICU of Sakarya University Education and Research Hospital between March 19 and April 13, 2020. The final date of the follow-up was April 18. Sakarya University Education and Research Hospital is a tertiary centre of the city with a capacity of 80 beds in ICU. The demographic characteristics, clinical data, and laboratory findings of all patients, who were diagnosed with Covid-19 according to the World Health Organisation interim guidance [7], were recorded. We excluded the patients under 18 years of age. The ethical approval was obtained from the local ethics committee. 

### 2.2. ICU admission criteria and mechanical ventilation indications

Patients with respiratory distress (>30 breaths/min), oxygen saturation < 90 at rest under nasal oxygenation with 5–6 litres/min, arterial partial pressure of oxygen (PaO2) / fraction of inspired oxygen (FiO2) < 300 mmHg presented to ICU. In addition to these, patients with sepsis (SOFA score greater than or equal to 2), immunosuppressed patients who have symptoms of shortness of breath, fever and/or cough, and significant comorbidities (chronic kidney disease, congestive heart failure, chronic obstructive pulmonary disease, and diabetes), which may significantly worsen with concomitant Covid-19 infection also presented to ICU. Mechanical ventilation indications are the presence of hypercapnic acidosis, hypoxemia despite administration of high flow nasal oxygen (FiO2 ≥ 60% and oxygen flow rate ≥ 40%), or severe dyspnoea with increased work (rate?)_of breathing (recruitment of accessory and expiratory muscles, intercostal recession, or nasal flaring). Lung protective ventilation strategy was applied for patients with ARDS who require mechanical ventilation. 

### 2.3. Specimen collection and testing

Only laboratory confirmed cases, which was defined by a positive result on a real-time reverse transcriptase polymerase chain reaction (PCR) assay of a specimen obtained from the patient, were included this study. To identify SARS-CoV-2 infection, respiratory specimens, including nasal and pharyngeal swabs, were collected from all patients at their presentation to the hospital and during their hospitalisation. Patients underwent blood tests including complete blood count, electrolytes, kidney and liver function tests, creatin kinase and MB fraction, high sensitive (hs) troponin, d-dimer, ferritin, C-reactive protein and procalcitonin. All patients also underwent computed tomography of the chest. The blood test parameters on the first day of admission to the ICU were analysed. 

### 2.4. Outcomes

The clinical outcomes (Covid-19 associated death, discharge from ICU, and length of stay in ICU) were recorded. Acute respiratory distress syndrome (ARDS) was defined according to the Berlin definition [8]. Acute kidney injury was identified according to the Kidney disease: improving global outcomes definition [9]. The multiple organ dysfunction syndrome (MODS) was defined as the development of potentially reversible physiologic derangement involving two or more organ systems [10].

### 2.5. Statistical analysis

Descriptive analysis of the variables were expressed as mean ± SD in normal distribution, and parameters with abnormal distribution were expressed as median of the 25th–75th percentile (interquartile range). Categorical data are expressed as proportions. The chi-square and the Student’s t-test were used for categorical and continuous variables, respectively. Fisher’s exact test was applied in analysing small samples. For continuous variables, differences between the two groups were evaluated using the Student’s t-test when data were normally distributed and the Mann–Whitney U test when the assumption of normality was not met. Binary logistic regression analysis was performed to determine independent factors associated with mortality. A P-value less than 0.05 was considered statistically significant. Statistical analyses were performed using statistical software (SPSS 20.0, IBM Corporation, Armonk, Chicago, IL, USA).

## 3. Results

### 3.1. Demographic and clinical features

A total of 103 patients were included in this study. The mean age of the patients was 69.6 ± 14.1 years (ranged between 24 to 95). Forty-four of the patients were female (42.7%). The most common symptoms were fatigue (96 [93.2%]), shortness of breath (94 [91.3%]), cough (91 [88.3%]), fever (54 [52.4%]), anosmia (16 [15.5%]), sore throat (15 [14.6%]) and diarrhoea (4 [3.9%]). Sixty-two of the patients (60.2%) had a history of hypertension, 46 (44.7%) had diabetes mellitus, 28 (27.2%) had coronary artery disease, 24 (23.3%) had chronic obstructive pulmonary disease, 10 (9.7%) had cerebrovascular disease, 7 (6.8%) had chronic renal disease, 6 (5.8%) had congestive heart failure, and 12 (11.7%) had malignities. 26 of the patients (25.2%) had no comorbidities (Table 1). All of the patients underwent computed tomographic assessment of the chest before admission to ICU and a total of 102 (99%) patients had typical findings compatible with Covid-19 pneumonia. 

**Table 1:** Clinical and demographic characteristics of the patients.

Characteristics	Patients(n = 103)	Discharged(n = 30)	Died (n = 52)	P
Age (years)	69.6 ± 14.1	65.2 ± 15.8	72.5 ± 12.5	0.023*
Female sex (n, %)	44 (42.7%)	13 (43.3%)	23 (44.2%)	0.937
Signs and symptoms				
Fever (n, %)	54 (52.4%)	18 (60%)	27 (51.9%)	0.479
Cough (n, %)	91 (88.3%)	28 (93.3%)	45 (86.5%)	0.343
Shortness of breath (n, %)	94 (91.3%)	28 (93.3%)	47 (90.4%)	0.645
Fatigue (n, %)	96 (93.2%)	29 ( 6.7%)	48 (92.3%)	0.427
Diarrhoea (n, %)	4 (3.9%)	2 (6.7%)	1 (1.9%)	0.551
Sore throat (n, %)	15 (14.6%)	7 (23.3%)	7 (13.5%)	0.252
Anosmia (n, %)	16 (15.5%)	5 (16.7%)	9 (17.3%)	0.941
Comorbidities				
Hypertension (n, %)	62 (60.2%)	17 (56.7%)	33 (63.5%)	0.543
Diabetes mellitus (n, %)	46 (44.7%)	12 (40%)	25 (48.1%)	0.479
Coronary artery disease (n, %)	28 (27.2%)	9 (30%)	13 (25%)	0.623
Cerebrovascular disease (n, %)	10 (9.7%)	1 (3.3%)	6 (11.5%)	0.414
Congestive heart failure (n, %)	6 (5.8%)	2 (6.7%)	2 (3.8%)	0.621
Chronic renal disease (n, %)	7 (6.8%)	0 (0.0%)	6 (11.5%)	0.081
Chronic obstructive pulmonary disease (n, %)	24 (23.3%)	9 (30%)	11 (21.2%)	0.369
Malignancy (n, %)	12 (11.7%)	1 (3.3%)	9 (17.3%)	0.084
APACHE II score	20 (15–25)	15 (13–20)	21 (18–28)	<0.001*
SOFA score	4 (3–6)	3 (3–4)	6 (4–8)	<0.001*

*P < 0.05. APACHE: acute physiologic assessment and chronic health evaluation, SOFA: the sequential organ failure assessment

### 3.2. Laboratory findings 

Table 2 represents the laboratory findings of the patients on ICU admission. Most of the patients (102, [99%]) had increased C-reactive protein at admission to ICU. About one-third of the patients had lymphocytopenia (34, [33%]) and one-quarter of patients had thrombocytopenia (24, [23.3%]). Forty-one of the patients (39.8%) demonstrated liver injury with elevated hepatic enzymes. Arterial lactate levels were elevated in 72 of the patients (69.9%). 43 of the patients (41.7%) had increased procalcitonin levels. We observed elevated high sensitive troponin levels in 40 of the patients (38.8%). D-dimer levels were high in most of the patients (85, [82.5%]) and serum ferritin levels were high in 76 of the patients (73.8%).

**Table 2:** Laboratory findings of the patients at admission to ICU.

Parameters	Patients(n = 103)	Discharged(n = 30)	Died(n = 52)	p
Hematologic				
White blood cells, x109/mL	8.7 (6.1–10.9)	7.5 (4.8–10)	8.7 (6.2–12)	0.030*
Neutrophils, x109/mL	7 (4.6–9.7)	5.4 (3.5–8.6)	7 (5–10.8)	0.016*
Lymphocytes, x109/mL	0.8 (0.5–1.1)	0.8 (0.4–1.1)	0.7 (0.4–1.1)	0.661
Platelets, x109/mL	195 (152–253)	222 (186–266)	179 (124–242)	0.002*
Haemoglobin, g/dL	12 (10–13)	11.8 (10.2–13)	11 (10–13)	0.762
Haematocrits, %	36.9 ± 5.3	36.4 ± 5.3	36.1 ± 5.1	0.826
Biochemical				
Urea, mg/dL	58 (34–89)	45.5 (28–71.5)	63 (40.5–92)	0.020*
Creatinine, mg/dL	0.9 (0.7–1.4)	0.7 (0.6–1)	1.1 (0.8–1.7)	0.001*
ALT, U/L	28 (18–40)	30 (21–49)	26 (17–36)	0.292
AST, U/L	42 (32–61)	44 (29–56)	42 (34–64)	0.534
Sodium, mmol/L	135 (132–139)	135 (132–138)	134 (131–139)	0.650
Potassium, mmol/L	4.1 (3.6–4.5)	4.1 (3.7–4.2)	4.2 (3.5–4.7)	0.280
Creatin kinase, U/L	140 (68–260)	99 (50–174)	193 (87–393)	0.001*
Creatin kinase-MB, U/L	18 (14–25)	16 (11–20)	19 (15–26)	0.005*
C-reactive protein, mg/L	111 (54–174)	69 (32–152)	138 (68–184)	0.011*
Procalcitonin, ng/L	0.3 (0.1–1.5)	0.2 (0.1–0.6)	0.6 (0.1–4.6)	<0.001*
High-sensitive troponin, ng/L	22 (8–100)	9.7 (4.9–29)	41.5 (17.5–497)	<0.001*
D-dimer, µgFEU/L	1400 (736–3680)	1045 (439–1785)	1585 (728–4362)	0.041*
Ferritin, µg/L	671 (252–1547)	667 (205–1234)	764 (268–1977)	0.302
Blood gas analysis				
pH	7.39 (7.34–7.43)	7.40 (7.37–7.44)	7.38 (7.32–7.43)	0.046*
PaO2, mmHg	60 (41–80)	69 (48–81)	62 (40–79)	0.281
PaCO2, mmHg	40 (35–48)	42 (36–48)	38 (33–44)	0.207
HCO3, mmol/L	24 (21–27)	25 (21–28)	23 (21–25)	0.009*
Lactate, mmol/L	2.1 (1.6–2.5)	2.1 (1.7–2.4)	2 (1.4–2.5)	0.843
O2 saturation, %	85 (69–92)	88 (79–92)	85 (69–91)	0.073*
PaO2/FiO2	107 (63–135)	114 (66 –133)	108 (53–126)	0.324

*P < 0.05. ALT: alanine aminotransferase, AST: aspartate aminotransferase.

When we compared the laboratory findings of the patients who had died or discharged, the discharged group had significantly lower procalcitonin and high-sensitive troponin levels (0.2 [0.1–0.6] vs 0.6 [0.1–4.6] ng/L, P < 0.001 and 9.7 [4.9–29] vs 41.5 [17.5–497] ng/L, P < 0.001, respectively). The discharged group had lower neutrophil counts (median 5.4 [3.5–8.6] vs 7 [5–10.8] x109/mL, P = 0.016), higher platelet counts ( 222 [186–266] vs 179 [124–242] x109/mL, P = 0.002), lower urea and creatinin levels (45.5 [28–71.5] vs 63 [40.5–92] mg/dL, P = 0.02 and 0.7 [0.6–1] vs 1.1 [0.8–1.7] mg/dL, P = 0.001, respectively), lower creatin kinase and creatin kinase-MB levels ( 99 [50–174] vs 193 [87–393] U/L, P = 0.001 and 16 [11–20] vs 19 [15–26] U/L, P = 0.005, respectively), lower C-reactive protein levels ( 69 [32–152] vs 138 [68–184] mg/L, P = 0.011) and lower d-dimer levels ( 1045 [439–1785] vs 1585 [728–4362] mgFEU/L, P = 0.041) (Table 2). 

**Table 3:** Treatment, complications, and clinical outcome of the patients.

Characteristic	Patients(n = 103)
Treatment	
Oxygen inhalation, n (%)	41 (39.8%)
Invasive mechanic ventilation, n (%)	62 (60.2%)
Discharged from ICU, n (%)	1 (1.6%)
Died, n (%)	47 (75.8%)
Remained in ICU, n (%)	14 (22.5%)
Vasopressor, n (%)	47 (45.6%)
Continuous renal replacement therapy, n (%)	21 (20.4%)
Blood purification treatment, n (%)	4 (3.9%)
Hydroxychloroquine, n (%)	103 (100%)
Favipiravir, n (%)	52 (50.5%)
Lopinavir/Ritonavir, n (%)	40 (38.8%)
Steroids, n (%)	63 (61.2%)
Tracheostomy, n (%)	2 (1.9%)
Prone positioning, n (%)	45 (43.6%)
Complications	
ARDS, n (%)	63 (61.2%)
Acute kidney injury, n (%)	31 (30.1%)
MODS, n (%)	52 (50.5%)
Clinical outcome	
Median length of stay in ICU (IQR), days	
All patients	7 (5–12)
Died	7 (4–10)
Discharged	6 (3.8–10.2)
Discharged from ICU, n (%)	30 (29.1%)
Died, n (%)	52 (50.5%)
Remained in ICU, n (%)	21 (20.4%)

ARDS: acute respiratory distress syndrome, MODS: multiple organ dysfunction 364 syndrome, ICU: intensive care unit. IQR: interquartile range.

### 3.3. Treatment, complications and clinical outcomes 

All of the patients received hydroxychloroquine for five days (loading dose of 400 mg BID and maintenance dose of 200 mg BID) and antiviral therapy (52 of the patients (50.5%) received favipiravir, loading dose of 1600 mg BID and maintenance dose of 600 mg BID) and 40 of the patients (38.8%) received lopinavir-ritonavir). 21 of the patients (20.4%) were given continuous kidney therapy. Blood purification treatment (HA-330, Jafron, Zhuhai, China) was used in 4 patients (3.9%) with severe Covid-19 who might have a cytokine storm syndrome. Twenty-nine of the patients (28.2%) developed bacterial coinfection and appropriate antibiotherapy was initiated. Forty-seven of the patients (45.6%) presented with persistent hypotension, requiring vasopressor agents. Two of the patients required tracheostomy, one of them survived and was discharged with good a neurologic condition. Twenty-two patients following at prone position were received neuromuscular blockage (Table 3).

Sixty-three patients (61.2%) developed ARDS and received steroid treatment (1 mg/kg for 5 days), 31 patients (30.1%) had acute kidney injury and 52 patients (50.5%) had MODS during ICU follow-up. All of the patients were intubated due to type I hypoxia and 62 patients (60.2%) received mechanical ventilation. Only one patient was extubated and discharged from ICU among these patients. Of the 62 patients, 47 (75.8%) had died and 14 (22.5%) were still receiving mechanical ventilation. Noninvasive ventilation was not performed to any patient due to the risk of transmitting the virus to the intensive care staff. As of April 18, of the 103 patients, 52 (50.5%) had died, 30 (29.1%) had been discharged from the ICU, 21 (20.4%) were still in the ICU. The 19 of the remained patients were still receiving mechanical ventilation. The median ICU stay was 7 (4–10) days in patients who had died and 6 (3.8–10.2) days in discharged patients from ICU. Compared with patients who had died, discharged patients were younger (65.2 ± 15.8 vs 72.5 ± 12.5 years, P = 0.023) and had significantly lower APACHE II and SOFA scores (15 [13–20] vs 21 [18–28], P < 0.001; and 3 [3-4] vs 6 [4–8], P < 0.001, respectively) (Table 3). 

Age, sex, hypertension, diabetes mellitus, coronary artery disease, chronic obstructive pulmonary disease, CRP, procalcitonin, d-dimer, ferritin, lymphocyte, haemoglobin, APACHE score, and SOFA score were included in the equation. SOFA score was found to be the only predictor for mortality (odds ratio = 1.982, 95% confidence interval = 1.070–3.634, P = 0.030) (Table 4).

**Table 4:** Independent factors associated with mortality using binary logistic regression analysis.

	Exp (B)(odds ratio)	95% confidence interval	P
Age	1.024	0.963–1.088	0.453
Sex	1.096	0.164–7.323	0.925
Hypertension	0.910	0.142–5.850	0.921
Diabetes mellitus	1.112	0.201–6.169	0.903
Coronary artery disease	4.313	0.654–28.458	0.129
Chronic obstructive pulmonary disease	0.643	0.081–5.119	0.676
CRP	1.010	0.998–1.022	0.088
Procalcitonin	2.058	0.920–4.605	0.079
D-dimer	1.000	1.000-1000	0.267
Ferritin	1.000	0.999–1.001	0.720
Lymphocyte	1.540	0.268–8.857	0.309
Haemoglobin	0.887	0.518–1.518	0.662
APACHE score	0.819	0.666–1.007	0.058
SOFA score	1.972	1.070–3.634	0.030*

APACHE: acute physiologic assessment and chronic health evaluation, SOFA: the sequential organ failure assessment.

## 4. Discussion 

In this cohort study, we described the clinical characteristics, treatment protocols and clinical outcomes among the critically ill patients with Covid-19. We included all consecutive patients with Covid-19 who were admitted to ICU in our hospital between March 19 and April 13, 2020. Our study also identified the risk factors associated with death in patients with Covid-19 in ICU. Previously, older age has been reported as an important predictor of mortality [11]. This current study confirmed that increased age was also associated with death due to Covid-19 in ICU. 

The previous reports showed that Covid-19 is a relatively mild condition in most of the affected cases but it can be severe and deadly in older individuals with comorbidities [12,13]. These patients require intensive care and have higher mortality rates, similar to published data from China [4]. The majority of the patients had one or more comorbidities in our study. Hypertension, diabetes mellitus and coronary artery disease were common in most of our cases. A multicentre cohort study which was conducted in China reported that comorbidities were present in nearly half of the patients. Hypertension was reported as the most common comorbidity, followed by diabetes mellitus and coronary artery disease in the same study [11]. These findings were also compatible with our study. 

The most common symptoms were reported as fever, cough, shortness of breath and fatigue in hospitalised patients in previous studies [11,14]. In our study, we observed that fatigue, shortness of breath, cough and fever were most common symptoms. Sore throat, anosmia, and diarrhoea were less frequently observed as initial symptoms in our study. According to the initial symptoms, there was no significant difference between the patients who were discharged and died. 

It has been reported that elevated d-dimer levels, IL-6, hs-troponin, lactate dehydrogenase and lymphopenia levels were commonly seen in severe Covid-19 illness. Additionally, higher SOFA score on admission was found to be associated with higher in-hospital death rates [11]. In our study, we found that procalcitonin, hs-troponin, d-dimer, and C-reactive protein levels were significantly higher at admission to ICU in patients who died compared to discharged patients. Admission platelet levels were significantly higher in discharged patients compared to the patients who died. APACHE II and SOFA are prognostic scores widely used to predict mortality in ICU patients. In our study, initial APACHE II and SOFA scores at admission were lower in discharged patients compared to patients who died.

According to the results of our study, the case fatality rate was 50.5%. This rate is similar to the reports among critically ill patients from other countries [13–15]. However, it should be taken into account that 21 patients still remained in the ICU at the time data was censored; so this rate may be an underestimate. The case fatality rate was 58% in patients over 65 years of age or older, and 35.3% in patients younger than 65 years of age in our study. Although many factors affecting mortality have been elucidated, there are still many more unknowns related to this disease. Our youngest patient who died was a 39- years old male and had no coexisting disease. However, the 92-year-old female patient with many comorbidities was discharged uneventfully although mechanical ventilator support was required. 

Continuous venovenous hemodiafiltration (CVVHDF) was especially chosen as a renal replacement modality in patients with acute renal failure in order to prevent hemodynamic instability. Anticoagulation was achieved with heparin in all patients. During the CVVHDF sessions, the heparin dose, which was given as a standard 10 units/kg/h, was updated to 15 units/kg/h due to frequent filter clotting, but still filter clotting occurred in some patients.

Extracorporeal blood purification therapy has been proven to eliminate inflammatory cytokines such as IL-6 and reduce systemic inflammatory response [16–18]. These theoretical findings lead the hypothesis that use of blood purification therapies could be beneficial in patients with cytokine storm due to Covid-19. We applied blood purification therapy in 4 patients with possible cytokine storm. During the blood purification treatment process, the inotropic agent requirement decreased and haemodynamic parameters improved, but all 4 patients had died on follow-up. A case was reported that blood purification therapy can contribute to recovery, however, we did not observe any improvement in mortality in our experience [19].

Covid-19 itself and the drugs used are reported to be related with cardiac complications [20]. In our study, we observed QT prolongation possible to hydroxychloroquine use in only 3 patients. The hydroxychloroquine treatment was immediately discontinued. No other cardiac complications were observed in the follow-up of these patients. Coronary angiography was performed in one patient with a preliminary diagnosis of acute coronary syndrome, but normal coronary arteries without any obstruction was observed. Three patients developed ventricular arrhythmia and sudden cardiac arrest during follow-up. These results were compatible with the other studies in the literature [21,22].

It is known that in intubated patients with severe ARDS, prone positioning improves oxygenation and decreases mortality [23]. During Covid-19 outbreak, it is reported that awake prone positioning may be useful to improve oxygenation and prevent ICU transfers [24]. Based on this information, awake prone positioning applied in 23 of the patients in the ICU and 8 of them survived without requiring mechanical ventilation. On the other hand, prone positioning was applied in 22 of the intubated patients with severe ARDS and just 2 of them survived.

During the outbreak, a coordinated response is needed to prepare health systems to meet this unprecedented challenge [25]. In our hospital, ICU admission criteria for Covid-19 were determined and all physicians in the clinics were informed. The patients with an oxygen saturation >90 at rest under nasal oxygenation with 2–3 L/min were followed up in the clinics with close monitorisation. Any deterioration in the clinical situation was detected immediately and transportation to ICU was provided for further management. This strategy helped us with the effective use of ICU capacity. 

There are many factors that play a role in mortality for COVID-19. Wang et al. reported that critically ill COVID-19 patients aged higher than 70 years old, had arrhythmia, or a SOFA score above 4 have a high risk of mortality, which is consistent to our study [26].

To our knowledge, this is the largest cohort study among critically ill patients with Covid-19 admitted to the ICU in Turkey. Covid-19 has high mortality rates and patients with older age and comorbidities may require care in the ICU. We found that procalcitonin, hs-troponin, d-dimer and C-reactive protein levels at admission were significantly higher in patients who died. Additionally, admission platelet levels were significantly lower in patients who died compared to the discharged patients. 

Our study has some limitations. Firstly, this study was conducted in a single centre and has relatively small number of patients. Secondly, 21 patients remained in ICU at the time of data censoring on April 18, 2020. For this reason, the outcomes of those patients were not known. 

## Informed consent

Sakarya University ethics committee approval date:10/04/2020, number:E.4026
